# Amphiphilic Poly-*N*-vinylpyrrolidone Nanoparticles as Carriers for Nonsteroidal, Anti-Inflammatory Drugs: Pharmacokinetic, Anti-Inflammatory, and Ulcerogenic Activity Study

**DOI:** 10.3390/pharmaceutics14050925

**Published:** 2022-04-24

**Authors:** Andrey Kuskov, Dragana Nikitovic, Aikaterini Berdiaki, Mikhail Shtilman, Aristidis Tsatsakis

**Affiliations:** 1 Department of Technology of Chemical Pharmaceutical and Cosmetic Substances, D. Mendeleev University of Chemical Technology of Russia, 125047 Moscow, Russia; a_n_kuskov@mail.ru; 2 Department of Biomaterials, D. Mendeleev University of Chemical Technology of Russia, 125047 Moscow, Russia; shtilmanm@yandex.ru; 3 Laboratory of Histology-Embryology, Medical School, Voutes Campus, University of Crete, 71003 Heraklion, Greece; berdiaki@uoc.gr; 4 Center of Toxicology Science & Research, Division of Morphology, Medical School, Voutes Campus, University of Crete, 71003 Heraklion, Greece; tsatsaka@uoc.gr

**Keywords:** poly-*N*-vinylpyrrolidone nanoparticles, indomethacin, anti-inflammatory effect, pharmacokinetics

## Abstract

Nanoparticles are increasingly utilized as drug delivery agents. Previously, we have developed a drug delivery system based on amphiphilic derivatives of poly-*N*-vinylpyrrolidone (PVP-OD4000) with excellent biocompatibility. In the current study, we assessed the pharmacokinetics, anti-inflammatory profile, and ulcerogenic potential of indomethacin (IMC)-loaded PVP-OD4000 nanoparticles compared to the free drug. Wistar male rats were utilized for a pharmacokinetics study and an anti-inflammatory study. Loaded IMC exhibited a slower elimination rate (*p* < 0.05) and a higher blood plasma concentration at 8 and 24 h after intraperitoneal injection compared with free IMC. In addition, decreased uptake of loaded IMC in the liver and kidney compared to free IMC (*p* < 0.05) was detected. Furthermore, PVP-OD4000 nanoparticles loaded with IMC showed an enhanced anti-inflammatory effect compared to free IMC (*p* < 0.05) in carrageenan-induced and complete Freund’s adjuvant-induced–(CFA) sub-chronic and chronic paw edema treatment (*p* < 0.01; *p* < 0.01). Notably, upon oral administration of loaded IMC, animals had a significantly lower ulcer score and Paul’s Index (3.9) compared to the free drug (*p* < 0.05). The obtained results suggest that IMC loaded to PVP nanoparticles exhibit superior anti-inflammatory activity in vivo and a safe gastrointestinal profile and pose a therapeutic alternative for the currently available NSAIDs’ administration.

## 1. Introduction

Nanoparticles are nanoscopic materials in the 1 to 500 nm size range that exhibit various structural characteristics, morphologies, or shapes. Due to their exceptional ability to penetrate biological structures, they are increasingly utilized as drug delivery agents, thus resolving unmet medical and pharmaceutical needs [1,2].

Previously, we have developed a drug delivery system based on amphiphilic derivatives of poly-*N*-vinylpyrrolidone (Amph-PVP) consisting of different molecular weight polymeric hydrophilic fragments linked into varying lengths of hydrophobic n-alkyl chains [3,4]. Notably, in aqueous media, these derivatives self-assemble into core-shell polymeric nanoparticles able to efficiently entrap a multitude of hydrophobic drugs of different molecular weights [3,5,6,7]. Likewise, PVPs have been used to modify liposomal membranes, increasing their stability [8]. Both in vitro and in vivo assessments of our Amph-PVP nanoparticles showed excellent biocompatibility and safe administration at even high doses [9,10,11,12], verifying, thus, their application potential. Notably, the utilization of some poly-*N*-PVP-based carriers have been approved for clinical studies [13].

Notably, various administration routes can be implemented to deliver nanocarrier–drug complexes, including oral administration, injection into blood vessels, inhalation, and transdermal application [14]. However, the efficacy of utilizing the respective routes as well as possible deleterious consequences regarding pharmacokinetics, tissue distribution, toxicity, disturbed homeostasis, and immunological response need to be evaluated [15,16,17,18].

The clearance of nanocarrier–drug complexes is of utmost significance as slow controlled clearance facilitates the delivery of the drug to target organs. Thus, the reticuloendothelial system (RES), a branch of the immune system composed of phagocytic cells, has been established as the key site of nanoparticle accumulation upon systemic administration. The liver, spleen, and lungs are the primary organs correlated with RES. Indeed, the liver has the highest ability for NP uptake [19]. Moreover, increased uptake and sequestration of nanoparticles in the liver attenuate the delivery to the targeted diseased tissue and potentially lead to enhanced toxicity at the hepatic tissue level [19].

Indomethacin (IMC), ((1-p-chlorobenzoyl)−5-methoxy-2-methylindole-3-acetic acid) is a nonsteroidal anti-inflammatory drug (NSAID) characterized by strong antipyretic, analgesic, and anti-inflammatory activity [20]. Even though it has an acceptable biodistribution, IMC, as a weak organic acid, is up to 90% bound to plasma proteins and thus binds into various tissue structures [21]. Indeed, due to its tight binding to plasma albumin, the available free fraction of the drug, similar to other NSAIDs, is decreased for brain uptake [22]. Likewise, the achievement of peak IMC concentration is delayed and decreased in synovial fluid compared to the plasma fraction [23]. Therefore, increasing IMC’s bioavailability without negatively affecting its biocompatibility is important in developing more efficient anti-inflammatory therapies.

The present study aimed to assess the in vivo performance of IMC-loaded PVP-OD4000 nanoparticles compared to free IMC under intraperitoneal and oral administration routes. The PVP-OD4000 amphiphilic polymer consists of a hydrophilic macromolecular fragment of PVP with 4000 Da molecular weight and one anchor hydrophobic n-alkyl octadecyl group (**5**). The PVP-OD4000 polymer was chosen, based on previous studies, as the most suitable candidate for achieving the optimal incorporation of hydrophilic and hydrophobic blocks, and bestowing nanoparticles with superior physicochemical properties, stability, and biocompatibility [5,11,12]. The anti-inflammatory effect of free IMC and IMC-loaded PVP-OD4000 nanoparticles was estimated in models of acute, sub-chronic, and chronic inflammation. Likewise, intraperitoneal and oral administration routes were utilized. Moreover, the blood and tissue pharmacokinetics of the free IMC and IMC loaded into the NP carrier system were estimated. The results of the present study suggest that IMC loaded to Amph-PVP exhibits superior anti-inflammatory activity in vivo, indicating that the PVP-OD4000 nanoparticles enhance the efficiency of this drug.

## 2. Materials and Methods

### 2.1. Materials

N-vinylpyrrolidone (VP), IMC, carrageenan, complete Freund’s adjuvant (CFA; heat treated and dried), and all other chemicals used in this study were obtained from Sigma (St. Luis, MO, USA) unless otherwise specified. The reagents were purchased at the available reagent grade and used without further purification. Specifically, all solvents and components of buffer solutions were of analytical quality and were utilized as received. All analytical reagents were HPLC-grade. Ultrapure water was prepared using Milli-Q Plus System (Millipore, Burlington, MA, USA).

### 2.2. Amphiphilic Polymer PVP-OD4000 Preparation

According to the method previously developed by our group, Amph-PVP polymer (PVP-OD4000) was synthesized by free-radical polymerization of N-vinylpyrrolidone monomer in the presence of azobisisobutyronitrile (AIBN) as initiator and mercaptoacetic acid (MAA) as chain-growth regulator. The following coupling reaction between carboxyl end-capped poly-*N*-vinylpyrrolidone with aliphatic n-octadecylamine (OD) in the presence of N, N’-dicyclohexylcarbodiimide (DCC) resulted in the desired polymer [24,25,26]. The resulting PVP-OD4000 amphiphilic polymer consists of a hydrophilic macromolecular fragment of PVP with 4000 Da molecular weight and one anchor hydrophobic n-alkyl octadecyl group. Due to its structure, such amphiphilic polymer can self-assemble in aqueous media at concentrations higher than critical aggregation concentration with the formation of micelle-like “corona-shell” type nanoparticles, consisting of the hydrophobic core formed by octadecyl groups due to hydrophobic interactions, and hydrophilic outer shell formed by PVP fragments (**5**).

The polymer purification was perpetrated by dialysis against ultrapure water using Slide-A-Lyzer Dialysis Cassettes (Thermo Fisher Scientific, Waltham, MA, USA) with a molecular weight cut-off (MWCO) of 1000 Da. The obtained medium was spectrophotometrically analyzed for impurities every 24 h, while its conductivity (µS/cm) was assessed at the 6 and 24 h points. The water was replaced with fresh water every 24 h. After three days of monitoring, no significant conductivity reduction or the presence of impurities was observed in the external solution, and the dialysis was terminated. The purified polymer solutions were in continuation freeze-dried (Martin Christ, Osterode am Harz, Germany) and used for further experiments.

Steam osmometry using vapor pressure osmometer (Knauer, Berlin, Germany) or alternatively titration were utilized to determine polymer average molecular weight. Size-exclusion chromatography (SEC) using a TSK Gel G4000PwxL column (Toso Co., Ltd., Shinkawa, Japan) was used to assess the polydispersity of the prepared polymers. Pyrene fluorescence probe spectrometry was applied to identify the critical aggregation concentration (CAC) of the PVP-OD4000 polymer [27,28].

### 2.3. Indomethacin-Loaded PVP-OD4000 Nanoparticles Preparation

The PVP-OD4000 nanoparticles containing IMC (C I8280, Sigma, St Luis, MO, USA) were prepared by the oil in water (O/W) emulsion method with further solvent evaporation [5].

Specifically, a small amount of chloroform was used to dissolve amphiphilic PVP-OD4000 and IMC (1.0:0.25 polymer–drug weight ratios). This mixture was then added drop-wise under intense stirring to the aqueous phase. The emulsification of the mix was achieved by ultrasonic treatment at 70 W HF power (Sonoplus HD 2070, Bandelin, Berlin, Germany). Then, the chloroform was eliminated under reduced pressure utilizing a rotary evaporator (Laborota 4010, Heidolph, Schwabach, Germany). Finally, the obtained IMC-loaded PVP-OD4000 nanoparticle suspensions were frozen and lyophilized by the Alpha I-4LD freeze dryer system (Martin Christ, Berlin, Germany) to yield dried nanoparticle products. The hollow PVP-OD4000 nanoparticles without IMC, used as placebo control, were prepared using the same method without IMC. Thermogravimetric analysis of freeze-dried nanoparticles confirmed that no residues of chloroform remained in the final preparations.

### 2.4. Characterization of PVP-OD4000 Nanoparticles

A dynamic light scattering (DLS) method using a light scattering spectrometer (Malvern Zetasizer Nano-ZS, Malvern, UK), at a wavelength of 633 nm, in PBS (pH 7.4) at 37 °C was utilized to determine the average hydrodynamic diameters and the size distribution of the IMC-loaded and hollow PVP-OD4000 nanoparticles. A scattering angle of 90° was duly used to determine the scattered laser intensity. All experiments evaluating the size of nanoparticles were performed in triplicate.

The zeta potential of the PVP-OD4000 nanoparticles was assessed utilizing a Zetasizer Nano-ZS (Malvern Instruments Ltd., Malvern, UK) in folded capillary cells. The surface charge of the PVP-OD4000 nanoparticles diluted suspensions in PBS (pH 7.4) was obtained for each nanoparticle sample three times, at which point the averages of these replicates were calculated. The zeta potential was calculated automatically by the device using the Smoluchowski equation

$$\zeta =\frac{\mu \times \eta }{\varepsilon },$$

where ζ is the zeta potential, *μ* is the mobility, *η* is the absolute viscosity of electrolyte solution, and *ε* is the dielectric constant [29].

The amount of IMC loaded in the PVP-OD4000 nanoparticles was measured using the Unico 2802 spectrophotometer (Unico, Dayton, NJ, USA) and monitored UV absorbance at 318 nm. The freeze-dried IMC-loaded PVP-OD4000 nanoparticles were preliminarily disrupted by adding ethanol and THF mixture (1:1, *v*/*v*) before IMC loading determination.

The content of IMC loaded into the hydrophobic core of the nanoparticles was determined from the amount of drug incorporated in nanoparticles and the total weight of drug-loaded nanoparticles using the following equation

$$\mathrm{IMC}\;\mathrm{content}\;\left(\%\right)=\frac{\mathrm{weight}\;\mathrm{of}\;\mathrm{IMC}\;\mathrm{in}\;\mathrm{nanoparticles}}{\mathrm{total}\;\mathrm{weight}\;\mathrm{of}\;\mathrm{IMC}\;\mathrm{loaded}\;\mathrm{nanoparticles}}\times 100$$

The drug loading efficiency (DLE) was evaluated using the following equation

$$\mathrm{DLE}\;\left(\%\right)=\frac{\mathrm{weight}\;\mathrm{of}\;\mathrm{entraped}\;\mathrm{IMC}\;\mathrm{in}\;\mathrm{nanoparticles}}{\mathrm{initial}\;\mathrm{weight}\;\mathrm{of}\;\mathrm{IMC}\;\mathrm{used}}\times 100$$

### 2.5. Characterization of IMC Release

Release studies were carried out at 37 °C under magnetic stirring. A total of 10 mL of phosphate buffer solution (PBS, 0.1 M, pH 7.4) with suspended IMC-loaded PVP-OD4000 nanoparticles were enclosed in a dialysis bag (1000 Da molecular weight cut-off), placed in 500 mL of PBS buffer at pH 7.4. At predetermined time intervals, 1.0 mL of buffer solution outside the dialysis bag was removed and replaced with 1.0 mL of fresh buffer solution. UV spectrophotometry (Unico, Dayton, NJ, USA) analyzed the amount of released IMC at 318 nm. Each result is an average of three parallel experiments.

### 2.6. Animals

Wistar rats (males, age: 7 to 8 weeks; body weight: 180–200 g) were acquired from the Branch of the M.M. Shemyakin and Yu. A. Ovchinnikov Institute of Bioorganic Chemistry of the Russian Academy of Sciences in Pushchino (Moscow Region, Russia). Animals were acclimatized for two weeks before the initiation of the experiments. The rats were housed in plastic cages (4 animals/cage) under conditions of controlled temperature maintained at 20–23 °C, the humidity of 60–70%, ventilation (10–12 times/h), and were maintained on a 12 h light/dark cycle. The animals were allowed free access to commercial food and water. All animal experiments were performed in compliance with the local regulations and guidelines on animal welfare and with EU Directive 2010/63/EU for animal experiments. Laboratory accreditation number ROSS RU.0001.21IM55 from 25 March 2016. The animals were humanely treated during the experiments, and no unnecessary stress was inflicted throughout.

### 2.7. Pharmacokinetics Study

Wistar rats (6 animals per group) were utilized to evaluate the nanoparticles’ pharmacokinetics profile. To obtain blood pharmacokinetic data, the rats received intraperitoneal injections of suspension of PVP-OD4000 nanoparticles loaded with IMC in the dose of 10 mg/kg BW and free IMC solution in the same dose. Blood samples were collected after specific time intervals, e.g., 0.5, 1, 4, and 8 h, up to 24 h after preparations’ injection. IMC concentration in rat blood plasma was determined using high-performance liquid chromatography (HPLC) as described [30].

The HPLC method was validated before the sample analysis. Specifically, Spectra Physics (Newport, CA, USA) chromatography, equipped with a LiChro-Cart RP-18 (column size 250 × 4 mm, 5 μm sorbent particle size) reverse-phase column and UV/VIS spectrophotometric detector Shimadzu SPD-M10AV (Kyoto, Japan), was used. The mobile phase consisted of methanol and 1% acetic acid (volume ratio 73:27, pH 3.0), with a flow rate of 1.0 mL/min. The IMC blood pharmacokinetics parameters were estimated using “Phoenix WinNonlin 5.3” software (Certara, Pheonix, NJ, USA). Group *t*-test (*p* = 0.05) was utilized to compare the statistical difference between the two groups.

The pharmacokinetics of tissues’ distribution was likewise evaluated utilizing Wistar rats (males, 180–200 g) as the study model. The animals were divided into two groups (6 animals per group). For this experiment, the PVP-OD4000 nanoparticle suspension containing a 10 mg/kg BW dose of IMC or free IMC solution with the exact dose were intraperitoneally injected into rats. After IMC preparations’ application, the animals were sacrificed at 8 h. In continuation, the organs (heart, lungs, liver, and kidneys) were excised, washed in saline solution, dried, and weighed. Then, the tissue samples were homogenized, and their extracts prepared. As described for the blood pharmacokinetics experiments, HPLC methods were utilized to determine IMC concentrations. Finally, the total amount of IMC accumulated in each tissue type after 8 h, respective to the mass unit of the tissue, was estimated. The *t*-test, with α = 0.05, considered statistically significant, was used to compare the difference in IMC tissue distribution between the two treatments.

### 2.8. Carrageenan-Induced Edema Acute Model

A carrageenan-induced rat paw edema model was used to assess the effect of IMC preparations on acute inflammation [31,32].

Wistar rats (males, 180–200 g) were divided into five groups (six rats per group). Group 1 served as control and received 0.2 mL of isotonic PBS solution (pH 7.4). Group 2 received 0.2 mL of PVP-OD4000 nanoparticle suspension without IMC and was designated as a placebo. Group 3 received 3.0 mg/kg BW of free IMC. Groups 4 and 5 received PVP-OD4000 nanoparticle suspensions with 1.0 mg/kg BW and 3.0 mg/kg BW of IMC, respectively. All preparations were administered as single intraperitoneal injections.

The above treatments were administered under two different treatment schedules, prophylactic and therapeutic. In the preventive scheme experiment, all treatments were applied preliminarily, 1 h before the subcutaneous injection of 0.05 mL of 1.0% carrageenan in saline solution (0.9%) into the rat’s right hind paw. In the therapeutic scheme experiment, the animals received the treatments 1 h after the carrageenan administration. Before the experiments, the rats fasted for 18 h with free access to drinking water, as the animal hydration state can influence the swelling intensity.

The right paw volume was determined half-hour before the carrageenan administration and 3 h after the injection using the plethysmometer device (Gemonio, Italy). The edema severity was then defined as the difference in the rat’s right hind paw volume before and after carrageenan application.

The inhibition of carrageenan inflammatory reaction in comparison to control was calculated in percentage for each animal using the following equation

$$I\;\left(\%\right)=\left(1-\frac{\Delta V_{treated}}{\Delta V_{control}}\right)\times 100,$$

where Δ*V_treated_* is the paw volume difference for preparations-treated animal groups (2–5) and Δ*V_control_* is paw volume difference for the control group 1.

### 2.9. Complete Freund’s Adjuvant-Induced Edema Sub-Chronic Model

A CFA-induced edema in a sub-chronic model was utilized to study IMC formulations effect [33,34].

Wistar rats were divided into four groups (six rats per group). All four animal groups received the subcutaneous injection of 0.1 mL CFA (0.5 mg/mL mycobacterium emulsion) in saline in the right hind paw to induce the edema.

In this sub-chronic model, rats were treated with different IMC preparations 2 h after the CFA injection and then every 24 h for 3 days. Group 1 served as control and was administered 0.2 mL of sterile saline. Group 2 received 0.2 mL of PVP-OD4000 nanoparticles suspension without IMC as a placebo. The animals in Group 3 were administered 3.0 mg/kg BW of free IMC. Group 4 received PVP-OD4000 nanoparticle suspension loaded with 3.0 mg/kg BW of IMC. All treatments were administered intraperitoneally.

The rat hind paw volumes were measured at 2, 4, 6, 8, 24, 48, and 72 h after CFA administration using the plethysmometer device (Gemonio Italy). The difference in edema volume in time was calculated and expressed in mL for these time points.

### 2.10. Complete Freund’s Adjuvant-Induced Arthritis Model

Twenty-four Wistar rats (males, 180–200 g) were randomly divided into four groups (six rats per group). The CFA-induced arthritis model used in the current study was previously described [35]. All four animal groups received the subcutaneous injection of 0.1 mL CFA (0.5 mg/mL mycobacterium emulsion) in saline in the right hind paw to induce the edema. All the rats were fed and had access to water normally after the induction of the inflammation. On day 14, all four animal groups started receiving appropriate preparation, two times a day, for 8 days, from the 14th day after CFA administration until the 21st day. Group 1 served as control and received 0.2 mL of sterile saline. Group 2 received 0.2 mL of PVP-OD4000 nanoparticle suspension without indomethacin as “placebo”. Group 3 received 3.0 mg/kg BW of free indomethacin. Group 4 received PVP-OD4000 nanoparticle suspension with 3.0 mg/kg BW of indomethacin. All preparations were injected intraperitoneally.

### 2.11. Ulcerogenic Activity Study

An ulcerogenic rat model was utilized as previously described to assess the putative ulcerogenic potential of free IMC and IMC-loaded PVP-OD4000 nanoparticles [36]. Specifically, twenty-four Wistar rats (males, 180–200 g) were fasted for 16 h before the experiment but had free access to water. The animals were randomly divided into four groups (six rats in each group). Group 1 received hollow PVP-OD4000 nanoparticles in PBS (pH 7.4) and was used as a negative control. Group 2 received free IMC at a dose of 40 mg/kg BW. Group 3 received IMC-loaded PVP-OD4000 nanoparticles corresponding to 40 mg/kg BW of IMC. Group 4 received IMC-loaded PVP-OD4000 nanoparticles corresponding to 60 mg/kg BW of IMC. All the preparations were administered orally by gavage through a metal tube attached to a 10 mL syringe, once a day for 4 days. On the 5th day, all rats were sacrificed with ether overdose.

Animal stomachs were extracted and dissected along the low curvature, rinsed with sterile saline (0.9%), and then macroscopically examined for signs of ulceration. A macroscopic assessment was made to evaluate the gastric mucosa’s state and determine the degree of the destructive lesion (the number of hemorrhages and erosions per animal). Moreover, the total length of destruction (pin-point hemorrhages, erosions, band-like lesions) was also estimated. Determination of ulcer scores was carried out according to an existing method [37]. Ulcers were independently assessed and scored by two pathologists using the following criteria—normal stomach (0), punctuate/pin-point ulcer (0.5), two or more small hemorrhagic ulcers (1), ulcers with a diameter larger than 2 mm (2).

Ulcers were scored, and the ulcer index value determined the ulcerogenic activity of tested preparations. Paul’s Index (PI) was used as the ulcer index [38] in this study.

The *PI* is an integral indicator of the extent of destruction in the stomach and is expressed as the formula

$$PI=\frac{M\times N}{100},$$

where *M* is the mean number of ulcers per rat in the group, *N* (%) is the percentage of rats with an ulcer.

### 2.12. Determination of Serum Cytokine Levels

Blood samples were collected from the CAF-induced arthritis model rats on the 21st day, immediately before their sacrifice. First, the blood was allowed to clot, and samples were centrifuged at 5 °C for 15 min at 1500 g. Then, the supernatant was frozen rapidly and stored at −70 °C for further measurements of interleukin-6 and -10 (IL-6, IL-10) and tumor necrosis factor (TNF-α) levels using enzyme-linked immunosorbent assay kits (ELISA), according to the manufacturer’s instructions (Biolegend, San Diego, CA, USA). The absorbance was read at 450 nm by an ELISA reader. The cytokines levels are expressed as pg/mL.

### 2.13. Statistical Analysis

Statistical significance was evaluated using a Student’s *t*-test, or one-way ANOVA analysis of variance. The results with *p* < 0.05 were considered statistically significant.

## 3. Results and Discussion

In our previous studies [5,6,12,26], we determined the IMC loading efficiency, capacity, and stability of Amph-PVP derivatives with a different molecular weight of polymer hydrophilic fragment and length of anchor long-chain aliphatic hydrophobic fragment. Furthermore, we assessed the in vitro IMC release performance and the cytotoxicity of these nanoparticles [11,12,26]. The respective studies demonstrated the excellent biocompatibility and performance of PVP nanoparticles, duplicated in an in vivo acute toxicity determination model.

Likewise, previously, the influence of a polymer structure on self-assembly processes in aqueous media as well as on the physicochemical properties of obtained polymeric nanoparticles was obtained [5,6]. Moreover, we optimized the efficiency of IMC entrapment and showed that it depended on both the polymer structure and the encapsulation conditions.

In the present study, we investigated in vivo the release profile and tissue distribution of IMC loaded to amphiphilic PVP nanoparticles to determine the efficacy of the new drug delivery system. Furthermore, we examined the activity of IMC loaded to PVP nanoparticles in different rat models of inflammation and compared this activity with the free drug. Moreover, the ulcerogenic effect in vivo of loaded with IMC PVP nanoparticles was assessed to determine their gastrointestinal safety.

For this purpose, three classical in vivo models of inflammation were utilized to estimate the short-term and long-term efficiency of IMC-loaded PVP-OD4000 nanoparticles in comparison to the free drug: carrageenan-induced acute inflammation, CFA-induced sub-chronic edema, and CFA-induced arthritis models.

### 3.1. IMC-Loaded PVP-OD4000 Nanoparticles Preparation and Characterization

**Figure 1:**
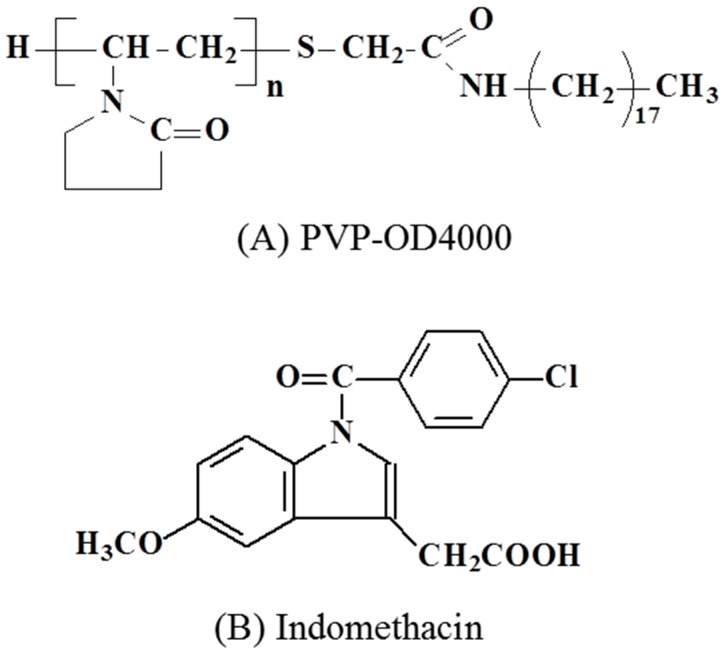
Structural formulas of n-octadecyl terminated N-vinylpyrrolidone amphiphilic polymer.

The amphiphilic PVP-OD4000 polymer, which consists of a hydrophilic PVP fragment with a molecular weight 4000 Da and one octadecyl hydrophobic group (Figure 1a), is able to self-assemble into nanoscaled aggregates. The said aggregates with high IMC entrapment efficiency, low in vitro cytotoxicity, low in vivo acute toxicity, and optimal in vitro sustained release profile were selected as the most probable drug carrier candidates for further in vivo investigations [25,27].

In the first stage, semitelechelic poly-*N*-vinylpyrrolidone with molecular weight 4000 Da, containing one terminal carboxyl group, was prepared by radical polymerization of VP monomer in the presence of the initiator (AIBN) and chain-growth regulator (MAA). In the second step, amphiphilic PVP derivative (PVP-OD4000) was obtained by a CDD-assisted coupling reaction between the semitelechelic PVP carboxyl group and aliphatic n-octadecyl amine.

The yield of the resulting polymer was 94%. The average molecular weight of the polymer determined by steam osmometry and, alternatively, by functional analysis (titration) matched in values was 4 kDa. The polydispersity index (PDI) of the prepared PVP-OD4000 polymer measured by SEC was 1.36.

As synthesized PVP-OD4000 polymer consists of large hydrophilic and hydrophobic blocks, it has the ability to self-assemble in aqueous media at concentrations higher than their critical aggregation concentration (CAC) to form nanoscaled spherical micelle-like associates with a hydrophobic inner core and a hydrophilic outer shell. The CAC of PVP-OD4000, determined by pyrene fluorescence probe spectrometry, was in the micromolar range (5.2 μM/L), i.e., sufficiently low for the “nanoparticles” stability and ability to maintain their self-assembled state during further experiments.

Previously, we showed that hydrophobic drugs, such as IMC (Figure 1b), are efficiently incorporated into the hydrophobic core during self-assembling of the amphiphilic PVPs into nanoscaled aggregates due to polymer–drug hydrophobic interactions [12].

Briefly, in this study, IMC-loaded PVP-OD4000 nanoparticles were prepared using single oil in water (O/W) emulsion. Hollow PVP-OD4000 nanoparticles without IMC were also obtained utilizing the same technique and were used in further experiments for comparison as a negative control or placebo. The freeze-dried IMC-loaded or hollow PVP-OD4000 nanoparticles were resuspended in PBS (pH 7.4) for further characterization and investigation. The composition and properties of the prepared nanoparticles are presented in Table 1.

The dynamic light scattering studies were performed in order to investigate the average hydrodynamic size, size distribution, and surface charge of the nanoparticles. The results obtained (Table 1) demonstrate that the average size of hollow PVP-OD4000 and IMC-loaded PVP-OD4000 nanoparticles was found to be smaller than 150 nm (124.7 ± 6.6 nm and 98.6 ± 4.9 nm, respectively), and their size distribution was narrow and monodisperse (PDI 0.132 ± 0.022 and 0.147 ± 0.036, respectively).

The IMC content in drug-loaded PVP-OD4000 nanoparticles and the drug loading efficiency (DLE) were determined spectrophotometrically. As shown (Table 1), the drug–polymer weight ratio of 0.25:1.0 bestows a high capability to entrap IMC (98.1%). The high leading efficiency allowed the preparation of the final PVP-OD4000 nanoparticles with approximately 20% (*w*/*w*) drug content.

**Figure 2:**
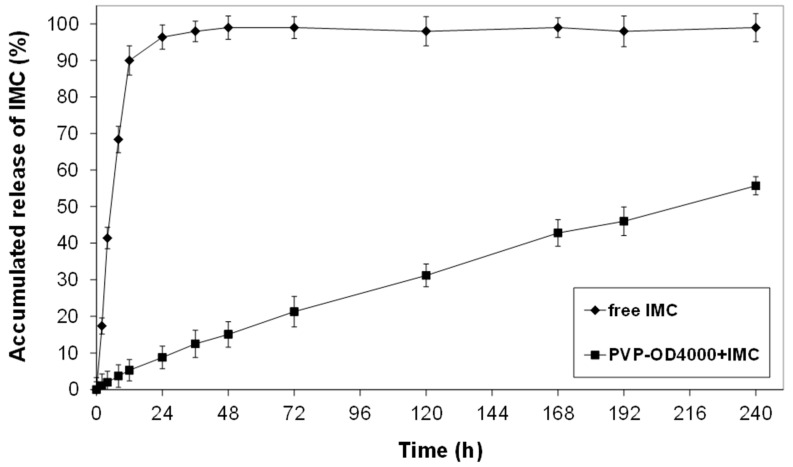
The release profile of IMC from PVP-OD4000 nanoparticles at pH 7.4 PBS and 37 °C.

The release of IMC from IMC-loaded PVP-OD4000 nanoparticles was investigated using dialysis membrane bags in phosphate buffer solutions (pH 7.4, 37 °C). Figure 2 demonstrates a release profile of IMC from PVP-OD4000 nanoparticles as a function of time. It is a plot of accumulated release as a % of the actual IMC load, determined from the loading efficiency.

As shown in Figure 2, while the free IMC exhibited rapid release of 98% within 24 h, the IMC, which was loaded into the inner hydrophobic core of PVP-OD4000 nanoparticles, demonstrated controlled release of about 52% for 10 days. This confirmed the hypothesis that IMC loaded into the nanoparticles’ core interacts hydrophobically with octadecyl groups of amphiphilic polymer, decreasing the drug release rate.

The release profile obtained for IMC-loaded PVP-OD4000 nanoparticles similar to our previous experiments for amphiphilic PVPs of various molecular sizes [5,6] showed that the major factor affecting the drug release rate is the binding affinity between the hydrophobic core of polymer nanoparticles formed by alkyl chains and the drug. Moreover, since no significant initial burst effect was observed from the release profile data, we can conclude that IMC is loaded into the PVP-OD4000 nanoparticles’ inner hydrophobic core without any residual drug bound on the surface.

Due to the large surface area/volume ratio, nanoparticles tend to agglomerate and adsorb proteins. Protein adsorption, however, enhances their uptake by macrophages enabling their clearance before reaching target cells [39]. The mechanism of protein adsorption has been attributed to electrostatic interaction, which can be controlled by variation in the surface charges [40,41].

Indeed, it has been previously demonstrated that nanoparticles with high surface charge and large particle size are phagocytized more efficiently by macrophages [42]. Furthermore, it has been established that scavenger receptors recognize highly negatively charged particles and facilitate their uptake by the RES [43]. At the same time, separate studies showed that highly negatively charged nanoemulsions are cleared faster and exhibit higher RES uptake than neutral or positively charged nanoemulsions [44]. However, positively charged micelles can exhibit enhanced cell uptake compared with negatively charged ones [45]. Indeed, it has been established that positively charged nanoparticles interact with the cell membrane’s negatively charged phospholipid components, enhancing nonspecific interactions with cells and circulating proteins [46]. Moreover, nanoparticles bearing a positive charge can initiate various undesirable biological responses such as the activation of the complement system and enhanced cytotoxicity [47]. Indeed, the impact of nanoparticle surface chemistry on nanoparticle–cell interactions has been firmly established [48]. Therefore, in this study, the nanoparticle surface charge, as a critical parameter that influences both nanoscaled carriers’ stability and in vivo behavior, was assessed by measuring zeta potential [40,41].

The zeta potential measurements for hollow and drug-loaded PVP-OD4000 are presented in Table 1. The two nanoparticle types were found to have mildly negative surface charges of 9.57 ± 0.79 mV and −7.15 ± 0.58 mV, respectively.

Previously, it was established that a low negative or positive charge of nanoparticles’ surface reduces their undesirable clearance by RES, such as the liver, and improves their blood compatibility, enhancing nanoparticle drug delivery ability [49,50]. Notably, it was also recently demonstrated that nanoparticles bearing the lowest absolute value of zeta potential can effectively avoid the uptake by MPS cells and thus exhibit a markedly delayed blood clearance [49,51]. Therefore, due to the undesirable effects of charged nanoparticle surfaces and increased clearance, it is important to decrease/neutralize the surface charge.

Thus, considering the characteristics of the determined PVP-OD4000 nanoparticles, e.g., low negative surface charge and mean sizes less than 150 nm, and the efficient encapsulation of IMC, it is suggested that these nanoparticles meet the requirements as prospective candidates for long-circulating, stable drug delivery systems for IMC.

### 3.2. In Vivo Pharmacokinetics and Biodistribution of Free IMC and IMC-Loaded-PVP-OD4000 Nanoparticles

**Figure 3:**
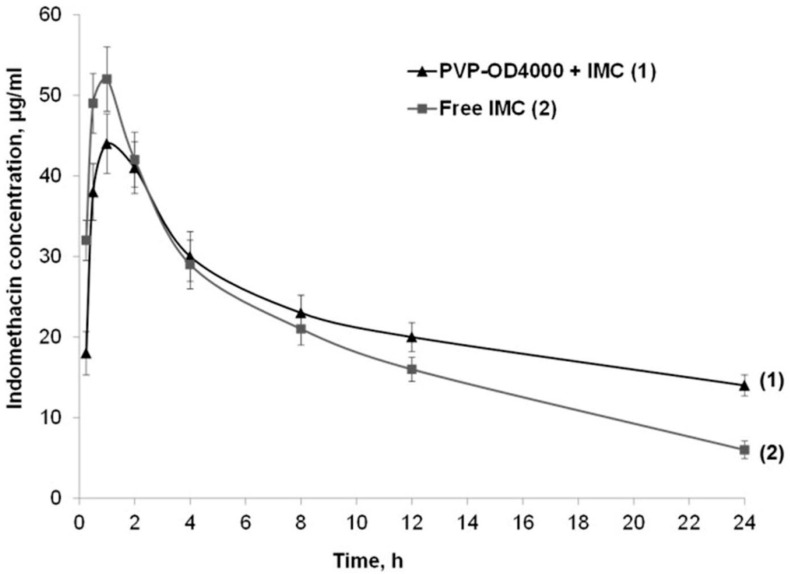
Indomethacin blood concentrations after single intraperitoneal administration of drug-loaded PVP-OD4000 nanoparticles and free drug preparation.

In continuation, blood and tissue pharmacokinetics of IMC incorporated into PVP-OD4000 and free IMC were assessed. Specifically, equal doses of IMC, either loaded to PVP-OD 4000 nanoparticles or in the form of a free drug suspension, were administered to rats as a single intraperitoneal injection. Subsequently, blood samples were collected at different time points, and IMC levels were determined (Figure 3). The obtained data demonstrate slightly different pharmacokinetic profiles of the two suspensions (Figure 3). Thus, the maximum IMC concentration levels for both preparations were achieved at 1 h after administration and estimated as 51.82 ± 6.47 μg/mL for free drug preparation and 43.93 ± 5.08 μg/mL for PVP-OD 4000 nanoparticle preparation. Notably, 12–24 h after the drug preparations’ administration, higher IMC blood levels were obtained in rats treated with IMC loaded with PVP-OD 4000 nanoparticles than those administered the free drug (*p* < 0.05).

Subsequently, the peak plasma concentration of a drug after administration (C_max_), time to reach C_max_ (t_max_), elimination rate constant (λ), area under the drug concentration curve (AUC), UC of (time × plasma drug concentration) versus time (AUCMC), the volume of distribution (VD), clearance (Cl), and mean residence time (MRT) were evaluated after the administration of both IMC preparations. As shown in Table 2, PVP-OD4000 nanoparticles exhibited a slower IMC elimination rate (*p* < 0.05) and, at the same time, higher AUC, AUMC, MRT, and distribution volume (VD) values in comparison with the free IMC preparation. These effects can be attributed to the controlled release of IMC from polymer nanocarriers and the PVP-OD4000 nanoparticles’ prolonged circulation time in vivo.

**Figure 4:**
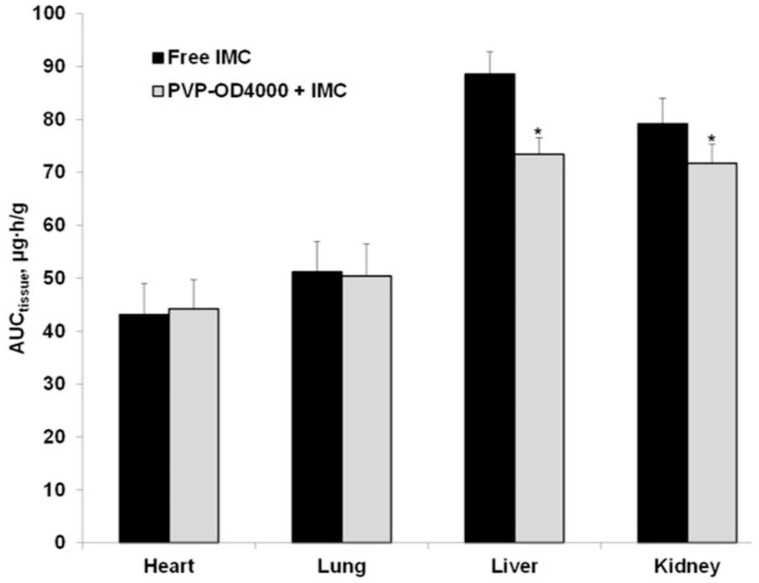
Indomethacin accumulation in each tissue type 8 h after single intraperitoneal administration of drug-loaded PVP-OD4000 nanoparticles and free drug preparation. * *p* < 0.05.

In continuation, the biodistribution of IMC, administered in the form of PVP-OD4000 nanoparticles and the free IMC solution, was assessed in various organ tissues 8 h after the drug administration. Specifically, concentrations of the drug concerning the mass unit of the heart, lung, liver, and kidney and total amounts of IMC accumulated in each type of organ for 8 h after both drug preparations’ application (AUC tissue) were determined (Figure 4).

This approach showed a lower distribution of entrapped IMC in PVP-OD4000 compared to free IMC in the kidney and liver (*p* < 0.05). Upon systemic administration, nanoparticles accumulated in RES, which has long been recognized as the major site of liposome accumulation. Indeed, the liver exhibits the largest capacity for liposome uptake [52]. At the same time, no significant difference was detected regarding lung and heart IMC distribution between the two suspensions. Thus, the observed decrease in entrapped IMC uptake by the kidney, the critical excretion organ, and the liver as the primary RES organ is well in agreement with the results of the blood pharmacokinetic studies, which demonstrated an increase in mean residence time (MRT) for IMC-loaded PVP-OD4000 nanoparticles compared to the free drug (11.61 ± 0.93 h to 21.66 ± 4.51 h).

To summarize, IMC-loaded amphiphilic PVP nanoparticles exhibit a superior bioavailability and biodistribution pharmacokinetic profile compared to free IMC. In addition, these traits support the prolonged controlled release of the drug.

### 3.3. The Effect of Free IMC and IMC-Loaded-PVP-OD4000 Nanoparticles on Carrageenan-Induced Acute Edema Model

The carrageenan administration to the rat hind paw is a commonly used model to study the acute inflammation process. Carrageenan injection leads to quick edema formation due to the exacerbated sensitivity to mechanical and thermal stimuli [53]. Indeed, the carrageenan-induced rat paw edema acute model is widely used to investigate new anti-inflammatory substances and drug formulations, including IMC, nanoparticle drug delivery systems, and mechanisms of their action [54,55].

In this study, IMC-loaded PVP nanoparticles and their free IMC formulation were injected intraperitoneally using two different administration schemes. The respective schemes were: prophylactic (before) and therapeutic (after) subcutaneous injection of edema-inducing carrageenan.

The results of this approach are presented in Table 3 and Table 4.

The prophylactic application of IMC preparations (free iIMC 3.0 mg/kg BW, PVP-OD4000 nanoparticles with IMC 3.0 mg/kg BW and PVP-OD4000 nanoparticles with INC 1.0 mg/kg BW) noticeably inhibited the carrageenan-induced edema in comparison with the control and placebo groups, with demonstrated inhibition of 57.0%, 79.5%, and 53.1%, respectively. Moreover, the acute therapeutic treatment using free IMC (3.0 mg/kg BW), PVP-OD4000 nanoparticles with indomethacin (3.0 mg/kg BW), and PVP-OD4000 nanoparticles with indomethacin (1.0 mg/kg BW), administered 1 h after carrageenan injection, resulted in edema inhibition of 33.2%, 70.7%, and 29.5%, respectively.

Notably, in both treatment schemes, drug-loaded amphiphilic PVP nanoparticles demonstrated higher anti-inflammatory activity than free IMC with equal drug content in preparations (3.0 mg/kg BW). Furthermore, even PVP-OD4000 nanoparticles with IMC content three times lower (1.0 mg/kg BW) showed an approximately equal inhibitory effect on carrageenan-induced edema compared to free indomethacin in a 3.0 mg/kg BW dose.

Our experimental approach confirms that PVP-OD4000 nanoparticles loaded with IMC show an enhanced anti-inflammatory effect compared with free IMC in carrageenan-induced paw edema treatment schemes. These results highlight the efficiency of amphiphilic PVP nanoparticles as carriers for IMC and other hydrophobic drugs.

### 3.4. The Effect of Free IMC and IMC-Loaded-PVP-OD4000 Nanoparticles on Complete Freund’s Adjuvant-Induced Edema Sub-Chronic Model

**Figure 5:**
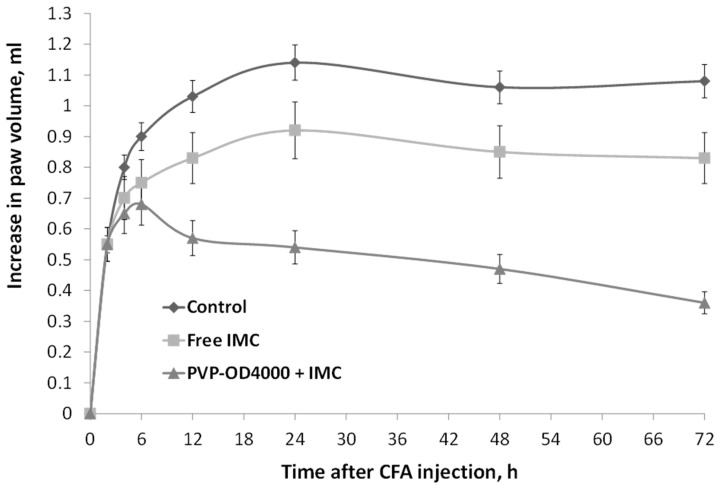
The effect of IMC-loaded PVP-OD4000 nanoparticles and free IMC formulation (drug dose 3.0 mg/kg BW, intraperitoneal administration, 2 h after CFA injection, and once a day for 3 days), on rat hind paw CFA-induced edema (sub-chronic model). IMC-loaded to PVP-OD4000 compared to control (*p* < 0.001) and compared to free IMC (*p* < 0.01).

As IMC-loaded PVP-OD4000 nanoparticles showed a prolonged release profile with increased mean residence time in vivo, investigating the efficacy of nanoparticular formulation in a long-term inflammation model was reasonable. For this purpose, the CFA-induced sub-chronic hind paw model of inflammation was chosen. Specifically, two hours after CFA injection and once every day for 3 days, the animals were treated either with IMC-loaded PVP-OD4000 nanoparticles (3 mg/kg BW), free IMC (3 mg/kg BW), or hollow PVP-OD4000 (placebo) intraperitoneally. The CFA-induced edema was measured at 6, 14, 24, 28, and 72 h under all treatment options. As shown in Figure 5, IMC loaded to PVP-OD4000 significantly attenuated edema formation compared to the control (*p* < 0.001) and compared to free IMC (*p* < 0.01). On the other hand, the hollow PVP-OD4000 nanoparticles’ treatment (placebo) did not exert any effect on CFA-induced edema growth compared to the control animal group (*p* = NS). Thus, the placebo data were not included in the graph.

These data agree with the previous data confirming that IMC-loaded PVP-OD4000 nanoparticles possess noticeably higher anti-inflammatory activity than free IMC substances.

### 3.5. The Effect of Free IMC and IMC-Loaded PVP-OD4000 Nanoparticles on Complete Freund’s Adjuvant-Induced Arthritis Model

The good anti-inflammatory activity of IMC-loaded amphiphilic PVP nanoparticles shown in experiments on acute and sub-chronic inflammation edemas, motivated us to investigate nanoparticular IMC formulation efficiency in a chronic inflammation model. For this purpose, we utilized a CFA-induced arthritis experimental model [56,57].

Specifically, upon the complete Freund’s adjuvant administration in the rat hind paw, local edema that is visible for up to 28 days develops. Moreover, CFA-induced edema in its later phase (after 14 days) is widely used as a model of arthritis [58].

In the current investigation, CFA-treated animals were administered intraperitoneally, free IMC formulation (3.0 mg/kg BW), IMC-loaded PVP-OD4000 nanoparticles (3.0 mg/kg BW), and hollow PVP-OD4000 for 8 days, two times a day, from the 14th up to the 21st day after the injection of CFA.

**Figure 6:**
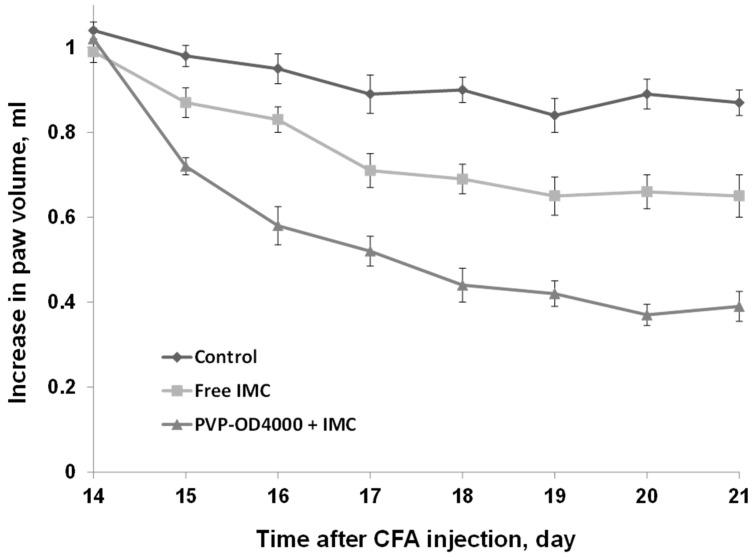
Influence of indomethacin-loaded PVP-OD4000 nanoparticles and free indomethacin formulation (3.0 mg/kg BW, intraperitoneally, for 8 days, two times a day, from 14th up to 21st day after the injection of CFA), on rat hind paw, induced arthritis model. IMC loaded to PVP-OD4000 compared to free IMC (*p* < 0.05) and compared to control (*p* < 0.001).

As shown in Figure 6, the repeated administration of both IMC-loaded PVP-OD4000 nanoparticles and free drug (between 14 and 21 days) attenuated CFA-induced long-term edema in rats. Moreover, amphiphilic PVP nanoparticles with entrapped IMC exhibited a more potent anti-inflammatory activity in arthritis model conditions than free IMC (*p* < 0.05) and compared to the control (*p* < 0.001), similar to previous experiments. On the other hand, hollow PVP-OD4000 nanoparticles did not exert any effect compared with the control animal.

The obtained results correlate well with improved drug pharmacokinetics, bioavailability, and biodistribution after immobilization in polymer nanoparticles, as also shown in other models [59,60].

In this particular case, PVP-OD4000 nanoparticles provide prolonged controlled release of IMC to the inflamed area in CFA-arthritis model experiments. It should also be mentioned that the maximum tested IMC dose loaded to amphiphilic PVP nanoparticles (3.0 mg/kg BW) is sub-therapeutic. Therefore, optimizing the new IMC nanoparticle dosage can further improve its anti-inflammatory activity.

### 3.6. The Effect of Free IMC and IMC-Loaded PVP-OD4000 Nanoparticles on Cytokine Release in Complete Freund’s Adjuvant-Induced Arthritis Model

Utilizing respective ELISA kits (Biolegend, San Diego, CA, USA), we measured serum IL6, -10, and TNF-α levels of blood samples taken on the 21st day of the CFA-induced arthritis group. The obtained results show that the treatment with IMC-loaded PVP-OD4000 nanoparticles (3.0 mg/kg BW of IMC, two times a day, for 8 days), from the 14th to the 21st day after CFA injection, induced significant inhibition in the production of IL-6 and TNF-α pro-inflammatory cytokines (Table 5). Moreover, the same treatment scheme with IMC-loaded PVP-OD4000 nanoparticles also increased IL-10 anti-inflammatory cytokine (Table 5). At the same time, treatment with free IMC preparation with equal IMC content (3.0 mg/kg BW) was less effective in modifying inflammatory markers (Table 5).

It is well established that the pro-inflammatory cytokines, including TNF-α, IL-1β, IL-17, and IL-6, are upregulated in the CAF-induced arthritis rat model [61,62]. Indeed, these cytokines’ production is an important component of the inflammatory mechanism and an excellent biomarker of disease progression [63].

The CAF-induced animals’ TNF-α, IL-6, and IL-10 levels (820 ± 96; 341 ± 34; 552 ± 57 pg/mL) were strongly upregulated compared to basal levels (29.6 ± 2.1; 31.2 ± 2.4; 26.2 ± 1.7 pg/mL) (*p* < 0.001), respectively. The data obtained show that treatment with the IMC-loaded PVP-OD4000 nanoparticles’ preparation was able to induce a significant decrease in IL-6 and TNF-α pro-inflammatory cytokines in the arthritis rat serum. At the same time, the application of the IMC-loaded PVP-OD4000 nanoparticles’ preparation caused a significant increase in the IL-10 anti-inflammatory cytokine serum levels. On the other hand, milder effects on cytokine production were determined when rats were treated with free IMC preparation at the same concentration. These data collectively demonstrate the superior anti-inflammatory effects of IMC loaded to PVP-OD4000 nanoparticles compared with free IMC.

### 3.7. Ulcerogenic Activity Study

Taking into account that the major side effect of most nonsteroidal anti-inflammatory drugs is a high risk of gastroduodenopathy [64,65], the last part of the current experimental study was dedicated to estimating the rat stomach mucosa conditions after daily intragastric administration of free and nanoparticular IMC formulations within four days.

**Figure 7:**
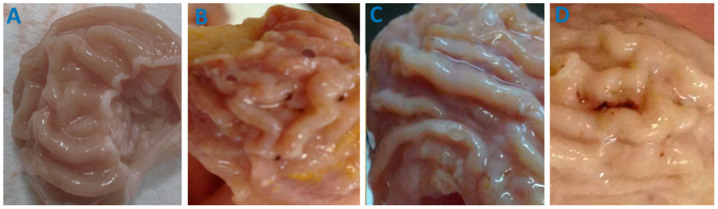
The effect of free and loaded to PVP-OD4000 IMC oral administration on rat stomach mucosa. (**A**) Hollow PVP-OD4000; (**B**) free IMC 40 mg/kg BW); (**C**) IMC loaded to PVP-OD4000 40 mg/kg BW; (**D**) IMC loaded to PVP-OD4000 60 mg/kg BW.

The rats were orally administered hollow PVP-OD4000 nanoparticles (group 1), free IMC 40 mg/kg BW (group 2), PVP-OD4000 nanoparticles containing 40 mg/kg BW of IMC (group 3), and PVP-OD4000 nanoparticles containing 60 mg/kg BW of IMC (group 4) during 4 days. On the 4th day, the animals were sacrificed, and animal stomachs were macroscopically examined. No harmful effects of hollow PVP-OD4000 on stomach mucosa were evident (Figure 7a). Macroscopic examination of group 2 rats (free IMC, 40 mg/kg BW) revealed noticeable mucous and serous membranes changes (Figure 7b). Specifically, marked hyperemia of the mucous membrane and multiple erosions with a diameter of 0.3 to 1.0 cm, filled with tissue detritus, were noted. Petechial hemorrhages with a diameter of 0.1 to 0.4 cm were also identified. Likewise, a serous membrane-hyperemia and a pronounced vascular pattern were determined (Figure 7b).

On the other hand, IMC-loaded PVP-OD4000 nanoparticles (40 mg/kg BW) showed low ulcerogenic activity. Specifically, the gastric mucosa was gray-pink, with well-contoured folds and no erosive defects evident. Mild focal hyperemia of the mucous membrane was identified. However, the serous membrane did not exhibit pathological findings (Figure 7c).

Stomachs of rats treated with IMC-loaded PVP-OD4000 nanoparticles (60 mg/kg BW) exhibited hyperemia of the mucous membrane as well as separate erosive defects with a diameter of 0.2 to 0.8 cm, filled with tissue detritus. Petechial hemorrhages with a diameter of 0.1 to 0.3 cm were also determined. In addition, hyperemia and a pronounced vascular pattern of the serous membrane were evident (Figure 7d). The quantification of the ulcerogenic activity was accomplished by determining the ulcer scores and Paul’s ulcer index (PI) (Table 6). Notably, group 3 animals had a significantly (*p* < 0.01) lower ulcer score (6.7 ± 1.94) and PI (3.9) in comparison to group 2 animals’, administered with the free drug (40 mg/kg BW), ulcer score (12.2 ± 1.31) and PI (12.8). Moreover, even the PVP-OD4000 nanoparticles’ preparation with IMC dose 1.5 times higher (60 mg/kg BW) (group 4) demonstrated significantly (*p* < 0.05) reduced gastrointestinal toxicity (ulcer score (10.1 ± 2.27) and PI (6.2)) in comparison to the free IMC group, after four days of daily administration.

The data in Table 6 show that the entrapment of IMC in the PVP-OD4000 nanoparticles’ core provides a significant ulcer formation reducing effect. Thus, animals treated with IMC loaded to PVP-OD4000 exhibit lower ulcer scores and PI than those treated with free IMC.

The presented data prove that IMC loaded to PVP nanoparticles induced significantly reduced stomach lesions compared to animals that received free IMC. Thus, IMC loaded to PVP nanoparticles has fewer side effects than the free drug, demonstrating increased gastrointestinal tolerance due to the controlled, sustained release of the active substance.

## 4. Conclusions

Amphiphilic PVP polymer (PVP-OD4000) was prepared with polydispersity. Due to its amphiphilic nature, this polymer can spontaneously self-assemble in aqueous media and form micelle-like nanoparticles with a spherical shape and mean diameter less than 150 nm. The nanoparticles exhibit a narrow size distribution, low negative surface charge, and can efficiently entrap IMC.

The evaluation of IMC-loaded PVP-OD4000 nanoparticles’ pharmacokinetics in vivo showed that the release profile of the drug exhibited a controlled and prolonged character. Furthermore, loading IMC to amphiphilic PVP nanoparticles protracted its circulation half-life in blood and decreased the drug uptake by the kidney and liver.

In the utilized models of acute carrageenan-induced, sub-chronic CFA-induced, and long-term CFA-induced arthritis inflammation, IMC-loaded amphiphilic PVP nanoparticles demonstrated enhanced anti-inflammatory efficiency compared with free drug formulation. Moreover, parameters of ulcerogenic activity indicate that IMC-loaded PVP-OD4000 nanoparticles exhibited increased gastrointestinal safety.

Thus, the data reported in this study clearly indicate that Amph-PVP self-assembled nanoparticles can successfully entrap IMC and deliver it to the inflammatory sites supporting its sustained release. Furthermore, the obtained results suggest that IMC loaded to Amph-PVP exhibit superior anti-inflammatory activity in vivo and a safe gastrointestinal profile, thus posing a therapeutic alternative for the currently available NSAIDs’ administration.

## Figures and Tables

**Table 1:** Characteristics of PVP-OD4000 nanoparticles (mean ± SEM, *n* = 3).

Nanoparticle Type	IMC/Polymer Weight Ratio	Particle Size (nm)	Particle PDI	Zeta Potential (mV)	IMC DLE (%)	IMC Content (%)
PVP-OD4000	0.0:1.0	124.7 ± 6.6	0.132 ± 0.022	−9.57 ± 0.79	-	-
IMC-loaded PVP-OD4000	0.25:1.0	98.6 ± 4.9	0.147 ± 0.036	−7.15 ± 0.58	98.1	19.6

**Table 2:** Comparison of main pharmacokinetic parameters of free IMC and polymer nanoparticles with entrapped indomethacin after single intraperitoneal administration.

Pharmacokinetic Parameter	Free IMC	PVP-OD4000 + IMC
C_max_ (μg/mL)	51.82 ± 6.47	43.93 ± 5.08
t_max_ (h)	0.9 ± 0.23	0.9 ± 0.23
λ (1/h) *	0.082 ± 0.009	0.054 ± 0.014
AUC (μg∙h/mL) *	516.70 ± 68.41	738.91 ± 110.16
AUMC (μg∙h^2^/mL) *	5982.74 ± 1026.09	16026.23 ± 6121.12
VD (mL/kg) *	202.63 ± 57.54	298.34 ± 39.89
Cl (mL/h)	15.05 ± 2.12	15.12 ± 2.03
MRT (h) *	11.61 ± 0.93	21.66 ± 4.51

The peak plasma concentration of a drug after administration (C_max_); time to reach C_max_ (t_max_); elimination rate constant (λ); area under the drug concentration curve (AUC); UC of (time × plasma drug concentration) versus time (AUCMC); volume of distribution (VD); clearance (Cl); mean residence time (MRT). * Significant difference (*p* < 0.05).

**Table 3:** Intraperitoneal acute prophylactic treatment effect of different IMC formulations on carrageenan-induced paw edema in rats.

Animal Group	Indomethacin Dose (mg/kg BW)	Paw Volume Difference (mL ± SEM ^a^)	Paw Volume Increase (%)	Edema Inhibition I (%)
Group 1 (Control)—PBS ^b^	0	0.250 ± 0.004	139.8	-
Group 2 (Placebo)—PVP-OD4000 nanoparticles	0	0.244 ± 0.009	136.5	-
Group 3—Free indomethacin	3.0	0.108 ± 0.005 **	60.1	57.0
Group 4—Indomethacin-loaded PVP-OD4000 nanoparticles	1.0	0.118 ± 0.007 **	65.6	53.1
Group 5—Indomethacin-loaded PVP-OD4000 nanoparticles	3.0	0.052 ± 0.006 **	28.7	79.5

^a^ SEM: standard error of the mean. ^b^ PBS: phosphate-buffered saline. ** *p* < 0.01 compared to controls.

**Table 4:** Intraperitoneal acute treatment effect of different IMC formulations on carrageenan-induced paw edema in rats.

Animal Group	Indomethacin Dose (mg/kg BW)	Paw volume Difference (mL ± SEM ^a^)	Paw Volume Increase (%)	Edema Inhibition I (%)
Group 1 (Control)—PBS ^b^	0	0.420 ± 0.006	208.6	-
Group 2 (Placebo)—PVP-OD4000 nanoparticles	0	0.425 ± 0.008	211.1	-
Group 3—Free indomethacin	3.0	0.278 ± 0.007 **	139.4	33.2
Group 4—Indomethacin-loaded PVP-OD4000 nanoparticles	1.0	0.294 ± 0.006 **	147.1	29.5
Group 5—Indomethacin-loaded PVP-OD4000 nanoparticles	3.0	0.122 ± 0.005 **	61.2	70.7

^a^ SEM: standard error of the mean. ^b^ PBS: phosphate-buffered saline. ** *p* < 0.01, compared to controls.

**Table 5:** The effect of PVP-OD4000 nanoparticles, free IMC, and IMC-loaded PVP-OD4000 nanoparticles on serum cytokine levels in the CFA-induced arthritis model (CAF).

	CAF-Induced	PVP-OD4000	Free IMC	PVP-OD4000 + IMC
TNF-α (mean ± SEM ^a^)	820 ± 96	786 ± 78	597 ± 92 *	123 ± 48 **
IL-6 (mean ± SEM ^a^)	341 ± 34	292 ± 38	203 ± 44 *	36 ± 18 **
IL-10 (mean ± SEM ^a^)	552 ± 57	621 ± 46	814 ± 141 *	1846 ± 122 **

^a^ SEM: standard error of the mean. * *p* < 0.05, compared to CAF-induced animals. ** *p* < 0.01, compared to CAF-induced animals.

**Table 6:** Ulcerogenic activity of different indomethacin formulations in rats after 4 days of daily treatment.

Animal Group	Indomethacin Dose (mg/kg BW)	Mean Ulcer Score ± SEM ^a^	Paul’s Index (PI)
Group 1—PVP-OD4000 nanoparticles	0	0	0
Group 2—Free indomethacin	40.0	12.2 ± 1.31	12.8
Group 3—Indomethacin-loaded PVP-OD4000 nanoparticles	40.0	6.7 ± 1.94 **	3.9
Group 4—Indomethacin-loaded PVP-OD4000 nanoparticles	60.0	10.1 ± 2.27 *	6.2

^a^ SEM: mean ± standard error with six rats per group. * *p* < 0.05, compared to free IMC group. ** *p* < 0.01, compared to free IMC group.

## Data Availability

Not applicable.

## References

[1] Henrich-Noack P., Nikitovic D., Neagu M., Docea A.O., Engin A.B., Gelperina S., Shtilman M., Mitsias P., Tzanakakis G., Gozes I. (2019). The blood-brain barrier and beyond: Nano-based neuropharmacology and the role of extracellular matrix. Nanomedicine.

[2] Taghizadehghalehjoughi A., Hacimuftuoglu A., Cetin M., Ugur A.B., Galateanu B., Mezhuev Y., Okkay U., Taspinar N., Taspinar M., Uyanik A. (2018). Effect of metformin/irinotecan-loaded poly-lactic-co-glycolic acid nanoparticles on glioblastoma: In vitro and in vivo studies. Nanomedicine.

[3] Tawfik M., Hadlak S., Götze C., Sokolov M., Kulikov P., Kuskov A., Shtilman M., Sahel B.A., Henrich-Noack P. (2021). Live in-vivo neuroimaging reveals the transport of lipophilic cargo through the blood-retina barrier with modified amphiphilic poly-*N*-vinylpyrrolidone nanoparticles. J. Biomed. Nanotechnol..

[4] Basyreva L.Y., Voinova E.V., Gusev A.A., Mikhalchik E.V., Kuskov A.N., Goryachaya A.V., Gusev S.A., Shtilman M.I., Velonia K., Tsatsakis A.M. (2020). Fluorouracil neutrophil extracellular traps formation inhibited by polymer nanoparticle shielding. Mater. Sci. Eng. C Mater. Biol. Appl..

[5] Kuskov A.N., Voskresenskaya A.A., Goryachaya A.V., Shtilman M.I., Spandidos D.A., Rizos A.K., Tsatsakis A.M. (2010). Amphiphilic poly-*N*-vinylpyrrolidone nanoparticles as carriers for nonsteroidal anti-inflammatory drugs: Characterization and in vitro controlled release of indomethacin. Int. J. Mol. Med..

[6] Kuskov A.N., Villemson A.L., Shtilman M.I., Larionova N.I., Tsatsakis M.A., Tsikalas I., Rizos A.K. (2007). Amphiphilic poly-*N*-vinylpyrrolidone nanocarriers with incorporated model proteins. J. Phys. Condens. Matter.

[7] Sharma A.K., Arya A., Sahoo P.K., Majumdar D.K. (2016). Overview of biopolymers as carriers of antiphlogistic agents for treatment of diverse ocular inflammations. Mater. Sci. Eng. C.

[8] Iamskov I.A., Kuskov A.N., Babievskii K.K., Berezin B.B., Kraiukhina M.A., Samoilova N.A., Tikhonov V.E., Shtil’man M.I. (2008). New liposomal forms of antifungal antibiotics, modified by amphiphilic polymers. Prikl. Biokhimiia Mikrobiol..

[9] Villemson A.L., Kuskov A.N., Shtilman M.I., Galebskaya L.V., Ryumina E.V., Larionova N.I. (2004). Interaction of polymer aggregates based on stearoyl-poly-*N*-vinylpyrrolidone with blood components. Biochemistry.

[10] Kuskov A., Selina O., Kulikov P., Imatdinov I., Balysheva V., Kryukov A., Shtilman M., Markvicheva E. (2021). Amphiphilic poly(N-vinylpyrrolidone) nanoparticles loaded with DNA plasmids encoding Gn and Gc glycoproteins of the Rift Valley Fever virus: Preparation and in vivo evaluation. ACS Appl. Bio Mater..

[11] Tsatsakis A., Stratidakis A.K., Goryachaya A.V., Tzatzarakis M.N., Stivaktakis P.D., Docea A.O., Berdiaki A., Nikitovic D., Velonia K., Shtilman M.I. (2019). In vitro blood compatibility and in vitro cytotoxicity of amphiphilic poly-*N*-vinylpyrrolidone nanoparticles. Food Chem. Toxicol..

[12] Berdiaki A., Perisynaki E., Stratidakis A., Kulikov P.P., Kuskov A.N., Stivaktakis P., Henrich-Noack P., Luss A.L., Shtilman M.M., Tzanakakis G.N. (2020). Assessment of Amphiphilic Poly-*N*-vinylpyrrolidone Nanoparticles’ Biocompatibility with Endothelial Cells in Vitro and Delivery of an Anti-Inflammatory Drug. Mol. Pharm..

[13] Giodini L., Re F.L., Campagnol D., Marangon E., Posocco B., Dreussi E., Toffoli G. (2017). Nanocarriers in cancer clinical practice: A pharmacokinetic issue. Nanomedicine.

[14] Li J., Xu W., Liang Y., Wang H. (2017). The application of skin metabolomics in the context of transdermal drug delivery. Pharmacol. Rep..

[15] Curlin M., Barbir R., Dabelic S., Ljubojevic M., Goessler W., Micek V., Zuntar I., Pavic M., Bozicevic L., Pavicic I. (2021). Sex affects the response of Wistar rats to polyvinyl pyrrolidone (PVP)-coated silver nanoparticles in an oral 28 days repeated dose toxicity study. Part. Fibre Toxicol..

[16] Sedyakina N., Kuskov A., Velonia K., Feldman N., Lutsenko S., Avramenko G. (2020). Modulation of entrapment efficiency and in vitro release properties of BSA-loaded chitosan microparticles cross-linked with citric acid as a potential protein-drug delivery system. Materials.

[17] Engin A.B., Nikitovic D., Neagu M., Henrich-Noack P., Docea A.O., Shtilman M.I., Golokhvast K., Tsatsakis A.M. (2017). Mechanistic understanding of nanoparticles’ interactions with extracellular matrix: The cell and immune system. Part. Fibre Toxicol..

[18] Neagu M., Piperigkou Z., Karamanou K., Engin A.B., Docea A.O., Constantin C., Negrei C., Nikitovic D., Tsatsakis A. (2017). Protein bio-corona: Critical issue in immune nanotoxicology. Arch. Toxicol..

[19] Zhang Y.N., Poon W., Tavares A.J., McGilvray I.D., Chan W.C.W. (2016). Nanoparticle-liver interactions: Cellular uptake and hepatobiliary elimination. J. Control. Release.

[20] Insel P., Molinoff P.B., Ruddon R.W. (1996). Analgesic-antipyretic and anti-inflammatory agents and drugs employed in the treatment of gout. The Pharmacologic Basis of Therapeutics.

[21] Emori H.W., Champion G.D., Bluestone R., Paulus H.E. (1973). Simultaneous pharmacokinetics of indomethacin in serum and synovial fluid. Ann. Rheum. Dis..

[22] Bannwarth B., Netter P., Lapicque F., Pere P., Thomas P., Gaucher A. (1990). Plasma and cerebrospinal fluid concentrations of indomethacin in humans. Relationship to analgesic activity. Eur. J. Clin. Pharmacol..

[23] Brune K., Glatt M., Graf P. (1976). Mechanisms of action of anti-inflammatory drugs. Gen. Pharmacol..

[24] Kuskov A.N., Luss A.L., Gritskova I.A., Shtilman M.I., Motyakin M.V., Levina I.I., Nechaeva A.M., Sizova O.Y., Tsatsakis A.M., Mezhuev Y.O. (2021). Kinetics and mechanism of synthesis of carboxyl-containing N-vinyl-2-pyrrolidone telehelics for pharmacological use. Polymers.

[25] Kulikov P.P., Goryachaya A.V., Luss A.L., Shtilman M.I., Kuskov A.N. (2017). Amphiphilic poly-*N*-vinyl-2-pyrrolidone: Synthesis, properties, nanoparticles. Polym. Sci. Ser. D.

[26] Yagolovich A., Kuskov A., Kulikov P., Kurbanova L., Bagrov D., Artykov A., Gasparian M., Sizova S., Oleinikov V., Gileva A. (2017). Amphiphilic Poly(N-vinylpyrrolidone) Nanoparticles Conjugated with DR5-specific antitumor cytokine DR5-B for targeted delivery to cancer cells. Pharmaceutics.

[27] Kuskov A.N., Shtilman M.I., Goryachaya A.V., Tashmuhamedov R.I., Yaroslavov A.A., Torchilin V.P., Tsatsakis A.M., Rizos A.K. (2007). Self-assembling nanoscaled drug delivery systems composed of amphiphilic poly-*N*-vinylpyrrolidones. J. Non-Cryst. Solids.

[28] Basu Ray G., Chakraborty I., Moulik S.P. (2006). Pyrene absorption can be a convenient method for probing critical micellar concentration (cmc) and indexing micellar polarity. J. Colloid. Interface Sci..

[29] Bayindir Z.S., Yuksel N. (2010). Characterization of niosomes prepared with various nonionic surfactants for paclitaxel oral delivery. J. Pharm. Sci..

[30] Sato J., Amizuka T., Niida Y., Umetsu M., Ito K. (1997). Simple, rapid and sensitive method for the determination of indomethacin in plasma by high-performance liquid chromatography with ultraviolet detection. J. Chromatogr. B Biomed. Sci. Appl..

[31] Winter C.A., Risley E.A., Nuss G.W. (1962). Carrageenin-induced edema in hind paw of the rat as an assay for antiiflammatory drugs. Proc. Soc. Exp. Biol. Med..

[32] Tratsk K.S., Campos M.M., Vaz Z.R., Filho V.C., Schlemper V., Yunes R.A., Calixto J.B. (1997). Anti-allergic effects and oedema inhibition caused by the extract of Drymis winteri. Inflamm. Res..

[33] Stein C., Millan M.J., Herz A. (1988). Unilateral inflammation of the hindpaw in rats as a model of prolonged noxious stimulation: Alterations in behavior and nociceptive thresholds. Pharmacol. Biochem. Behav..

[34] Fehrenbacher J.C., Vasko M.R., Duarte D.B. (2012). Models of inflammation: Carrageenan- or complete Freund’s Adjuvant (CFA)-induced edema and hypersensitivity in the rat. Curr. Protoc. Pharmacol..

[35] Lorton D., Lubahn C., Engan C., Schaller J., Felten D.L., Bellinger D.L. (2000). Local application of capsaicin into the draining lymph nodes attenuates expression of adjuvant-induced arthritis. Neuroimmunomodulation.

[36] Adinortey M.B., Galyuon I.K., Asamoah N.O. (2013). Trema orientalis Linn. Blume: A potential for prospecting for drugs for various uses. Pharmacogn. Rev..

[37] Alphin R.S., Ward J.W. (1967). Actions of hexopyrronium bromide on gastric secretion in dogs and on gastric secretion and ulceration in rats. Arch. Int. Pharmacodyn. Ther..

[38] Martín-Aragón S., Benedí J., Villar A. (1994). Studies on the Antiinflammatory and Antiulcerogenic Activities of Tuberaria lignosa Extracts in Experimental Animals. Int. J. Pharmacogn..

[39] Lee A.L., Wang Y., Cheng H.Y., Pervaiz S., Yang Y.Y. (2009). The co-delivery of paclitaxel and Herceptin using cationic micellar nanoparticles. Biomaterials.

[40] Honary S., Zahir F. (2013). Effect of Zeta Potential on the Properties of Nano-Drug Delivery Systems—A Review (Part 1). Trop. J. Pharm. Res..

[41] Honary S., Zahir F. (2013). Effect of Zeta Potential on the Properties of Nano-Drug Delivery Systems—A Review (Part 2). Trop. J. Pharm. Res..

[42] He C., Hu Y., Yin L., Tang C., Yin C. (2010). Effects of particle size and surface charge on cellular uptake and biodistribution of polymeric nanoparticles. Biomaterials.

[43] Rigotti A., Acton S.L., Krieger M. (1995). The class B scavenger receptors SR-BI and CD36 are receptors for anionic phospholipids. J. Biol. Chem..

[44] Khan M.K., Nigavekar S.S., Minc L.D., Kariapper M.S., Nair B.M., Lesniak W.G., Balogh L.P. (2005). In vivo biodistribution of dendrimers and dendrimer nanocomposites—Implications for cancer imaging and therapy. Technol. Cancer Res. Treat..

[45] Mailander V., Landfester K. (2009). Interaction of nanoparticles with cells. Biomacromolecules.

[46] Verma A., Stellacci F. (2010). Effect of surface properties on nanoparticle-cell interactions. Small.

[47] Fornaguera C., Caldero G., Mitjans M., Vinardell M.P., Solans C., Vauthier C. (2015). Interactions of PLGA nanoparticles with blood components: Protein adsorption, coagulation, activation of the complement system and hemolysis studies. Nanoscale.

[48] Nel A.E., Madler L., Velegol D., Xia T., Hoek E.M., Somasundaran P., Klaessig F., Castranova V., Thompson M. (2009). Understanding biophysicochemical interactions at the nano-bio interface. Nat. Mater..

[49] Duan X., Li Y. (2013). Physicochemical characteristics of nanoparticles affect circulation, biodistribution, cellular internalization, and trafficking. Small.

[50] Xu F., Yuan Y., Shan X., Liu C., Tao X., Sheng Y., Zhou H. (2009). Long-circulation of hemoglobin-loaded polymeric nanoparticles as oxygen carriers with modulated surface charges. Int. J. Pharm..

[51] Xiao K., Li Y., Luo J., Lee J.S., Xiao W., Gonik A.M., Agarwal R.G., Lam K.S. (2011). The effect of surface charge on in vivo biodistribution of PEG-oligocholic acid based micellar nanoparticles. Biomaterials.

[52] Rattan R., Bhattacharjee S., Zong H., Swain C., Siddiqui M.A., Visovatti S.H., Kanthi Y., Desai S., Pinsky D.J., Goonewardena S.N. (2017). Nanoparticle-macrophage interactions: A balance between clearance and cell-specific targeting. Bioorg. Med. Chem..

[53] Rocha A.C., Fernandes E.S., Quintao N.L., Campos M.M., Calixto J.B. (2006). Relevance of tumour necrosis factor-alpha for the inflammatory and nociceptive responses evoked by carrageenan in the mouse paw. Br. J. Pharmacol..

[54] Quintao N.L.M., Medeiros R., Santos A.R.S., Campos M.M., Calixto J.B. (2005). The effects of diacerhein on mechanical allodynia in inflammatory and neuropathic models of nociception in mice. Anesth. Analg..

[55] Kawamura M., Hatanaka K., Saito M., Ogino M., Ono T., Ogino K., Matsuo S., Harada Y. (2000). Are the anti-inflammatory effects of dexamethasone responsible for inhibition of the induction of enzymes involved in prostanoid formation in rat carrageenin-induced pleurisy?. Eur. J. Pharmacol..

[56] van den Berg W.B., Joosten L.A., Helsen M., van de Loo F.A. (1994). Amelioration of established murine collagen-induced arthritis with anti-IL-1 treatment. Clin. Exp. Immunol..

[57] Corvo M.L., Boerman O.C., Oyen W.J., Jorge J.C., Cruz M.E., Crommelin D.J., Storm G. (2000). Subcutaneous administration of superoxide dismutase entrapped in long circulating liposomes: In vivo fate and therapeutic activity in an inflammation model. Pharm. Res..

[58] Liu Y.L., Lin H.M., Zou R., Wu J.C., Han R., Raymond L.N., Reid P.F., Qin Z.H. (2009). Suppression of complete Freund’ adjuvant-induced adjuvant arthritis by cobratoxin. Acta Pharmacol. Sin..

[59] Mora-Huertas C.E., Fessi H., Elaissari A. (2010). Polymer-based nanocapsules for drug delivery. Int. J. Pharm..

[60] Couvreur P., Barratt G., Fattal E., Legrand P., Vauthier C. (2002). Nanocapsule technology: A review. Crit. Rev. Ther. Drug Carr. Syst..

[61] Sultana F., Rasool M. (2015). A novel therapeutic approach targeting rheumatoid arthritis by combined administration of morin, a dietary flavanol and non-steroidal anti-inflammatory drug indomethacin with reference to pro-inflammatory cytokines, inflammatory enzymes, RANKL and transcription factors. Chem. Biol. Interact..

[62] Alaaeldin E., Abou-Taleb H.A., Mohamad S.A., Elrehany M., Gaber S.S., Mansour H.F. (2021). Topical Nano-Vesicular Spanlastics of Celecoxib: Enhanced Anti-Inflammatory Effect and Down-Regulation of TNF-alpha, NF-small ka, CyrillicB and COX-2 in Complete Freund’s Adjuvant-Induced Arthritis Model in Rats. Int. J. Nanomed..

[63] Meyer O. (2003). Role of TNF-alpha and cytokines in the physiopathology of rheumatoid arthritis. Therapeutic perspectives. Bull. Acad. Natl. Med..

[64] Beck P.L., Xavier R., Lu N., Nanda N.N., Dinauer M., Podolsky D.K., Seed B. (2000). Mechanisms of NSAID-induced gastrointestinal injury defined using mutant mice. Gastroenterology.

[65] Asako H., Kubes P., Wallace J., Gaginella T., Wolf R.E., Granger D.N. (1992). Indomethacin-induced leukocyte adhesion in mesenteric venules: Role of lipoxygenase products. Am. J. Physiol..

