# Correction: Kuskov et al. Amphiphilic Poly-*N*-vinylpyrrolidone Nanoparticles as Carriers for Nonsteroidal, Anti-Inflammatory Drugs: Pharmacokinetic, Anti-Inflammatory, and Ulcerogenic Activity Study. *Pharmaceutics* 2022, *14*, 925

**DOI:** 10.3390/pharmaceutics17030369

**Published:** 2025-03-14

**Authors:** Andrey Kuskov, Dragana Nikitovic, Aikaterini Berdiaki, Mikhail Shtilman, Aristidis Tsatsakis

**Affiliations:** 1 Department of Technology of Chemical Pharmaceutical and Cosmetic Substances, D. Mendeleev University of Chemical Technology of Russia, 125047 Moscow, Russia; a_n_kuskov@mail.ru; 2 Department of Biomaterials, D. Mendeleev University of Chemical Technology of Russia, 125047 Moscow, Russia; shtilmanm@yandex.ru; 3 Laboratory of Histology-Embryology, Medical School, Voutes Campus, University of Crete, 71003 Heraklion, Greece; berdiaki@uoc.gr; 4 Center of Toxicology Science & Research, Division of Morphology, Medical School, Voutes Campus, University of Crete, 71003 Heraklion, Greece; tsatsaka@uoc.gr

**Error in Figure**

In the original publication [1], there was a mistake in Figure 7 as published. The wrong image for Figure 7A was included. The corrected Figure 7 appears below. The authors state that the scientific conclusions are unaffected. This correction was approved by the Academic Editor. The original publication has also been updated.

**Figure 7:**
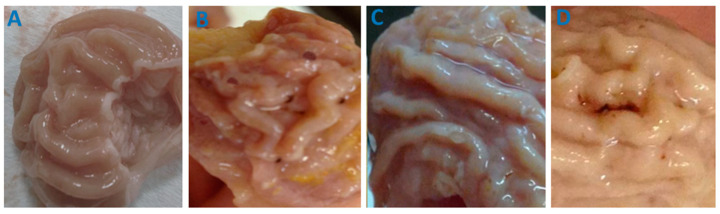
The effect of free and loaded to PVP-OD4000 IMC oral administration on rat stomach mucosa. (**A**) Hollow PVP-OD4000; (**B**) free IMC 40 mg/kg BW); (**C**) IMC loaded to PVP-OD4000 40 mg/kg BW; (**D**) IMC loaded to PVP-OD4000 60 mg/kg BW.

## References

[1] Kuskov A., Nikitovic D., Berdiaki A., Shtilman M., Tsatsakis A. (2022). Amphiphilic Poly-*N*-vinylpyrrolidone Nanoparticles as Carriers for Nonsteroidal, Anti-Inflammatory Drugs: Pharmacokinetic, Anti-Inflammatory, and Ulcerogenic Activity Study. Pharmaceutics.

